# Effectiveness of a Randomized Controlled Lifestyle Intervention to Prevent Obesity among Chinese Primary School Students: CLICK-Obesity Study

**DOI:** 10.1371/journal.pone.0141421

**Published:** 2015-10-28

**Authors:** Fei Xu, Robert S. Ware, Eva Leslie, Lap Ah Tse, Zhiyong Wang, Jiequan Li, Youfa Wang

**Affiliations:** 1 Nanjing Municipal Center for Disease Control and Prevention, Nanjing, China; 2 Department of Epidemiology and Biostatistics, School of Public Health, Nanjing Medical University, Nanjing, China; 3 School of Population Health, The University of Queensland, Brisbane, Queensland, Australia; 4 School of Health Sciences, Flinders University, Adelaide, South Australia, Australia; 5 School of Public Health and Primary Care, The Chinese University of Hong Kong, Hong Kong, China; 6 Department of Epidemiology and Environmental Health, School of Public Health and Health Professions, University at Buffalo, State University of New York, Buffalo, New York, United States of America; University, ITALY

## Abstract

**Background:**

Childhood obesity has been increasing rapidly worldwide. There is limited evidence for effective lifestyle interventions to prevent childhood obesity worldwide, especially in developing countries like China. The objective of this study was to assess the effectiveness of a school-based multi-component lifestyle childhood obesity prevention program (the CLICK-Obesity study) in Mainland China.

**Methods:**

A cluster randomized controlled trial was developed among grade 4 students from 8 urban primary schools (638 students in intervention, 544 as control) in Nanjing City, China. Students were randomly allocated to the control or intervention group at school-level. A one-year multi-component intervention program (classroom curriculum, school environment support, family involvement and fun programs/events) together with routine health education was provided to the intervention group, while the control group received routine health education only. The main outcome variables assessed were changes in body mass index, obesity occurrence, obesity-related lifestyle behaviors and knowledge.

**Results:**

Overall, 1108 (93.7%) of the 1182 enrolled students completed the intervention study. The intervention group had a larger marginal reduction than did the control group in overall mean BMI value (-0.32±1.36 *vs*. -0.29±1.40, p = 0.09), although this was not significant. Compared with the control group, the intervention group was more likely to decrease their BMI (OR = 1.44, 95%CI = 1.10, 1.87) by 0.5 kg/m^2^ or above, increase the frequency of jogging/running (OR = 1.55, 95%CI = 1.18, 2.02), decrease the frequency of TV/computer use (OR = 1.41, 95%CI = 1.09, 1.84) and of red meat consumption (OR = 1.50, 95%CI = 1.15, 1.95), change commuting mode to/from school from sedentary to active mode (OR = 2.24, 95%CI = 1.47, 3.40), and be aware of the harm of selected obesity risk factors.

**Conclusions:**

The school-based lifestyle intervention program was practical and effective in improving health behaviors and obesity-related knowledge for children in China. This study provides important policy implications on school-based intervention programs for modifications of obesity-related lifestyles.

**Trial Registration:**

Chinese Clinical Trial Registry ChiCTR-ERC-11001819

## Introduction

Childhood obesity has become a serious public health problem in both developed countries and developing societies including China [1–5]. The rising epidemic is largely attributed to unhealthy lifestyles and changes in the living environment during recent decades, for example, high consumption of high-density energy foods and reduced physical activity and/or increased sedentary behaviors [5–7]. Reducing childhood obesity requires effective lifestyle and behavior interventions that target healthy eating and physical activity (HEPA) among the general child population.

Most intervention studies regarding lifestyle and behavior modifications among children are school-based trials that have been conducted in Western societies with very few relevant studies in China [8–12]. Given a rapid epidemiological transition of lifestyle and behavior [13, 14] and increasing childhood obesity prevalence in China [15, 16], it is particularly important to develop effective and sustainable lifestyle and behavior intervention strategies for childhood obesity prevention among the general children population in China.

We developed a school-based multi-component lifestyle intervention, the CLICK-Obesity study, which aimed to reduce childhood obesity in China. It was designed to evaluate whether the lifestyle intervention was able to reduce obesity risk and increase healthy behaviors and knowledge. This report presents results regarding the effectiveness of the intervention.

## Methods

### Study design, participants and intervention components

#### 1) Study design

The CLICK-Obesity study was a school-based lifestyle and behavior intervention trial conducted within one academic school year (two semesters from September 2010 to June 2011) among fourth grade primary school students in Jianye, an urban district of Nanjing, China. Randomization occurred at the school level. Eight schools were randomly selected from thirteen primary schools within Jianye district based on estimates of sample size required and the average class size for primary schools. These eight selected schools were sorted according to the alphabetical order of their names from 1 up to 8, and then randomly allocated by the research team to receive either routine health education practice (4 schools) or the lifestyle and behavior intervention plus the routine health education program (4 schools) based on random numbers generated by software of Epical 2000. Full descriptions of the study design, participant eligibility criteria, intervention components, sample size calculations and preliminary findings from baseline data have been reported elsewhere [17, 18].

This study was approved by the academic and ethical committee of Nanjing Municipal Center for Disease Control and Prevention (Nanjing CDC) and was registered with the Chinese Clinical Trial Registry (ChiCTR-ERC-11001819).

#### 2) Study subjects

All the fourth graders within the eight chosen schools were eligible to participate (n = 1125). Written informed consent regarding baseline and follow-up surveys as well as participation in the lifestyle intervention were obtained from parents/guardians and the schools prior to the baseline survey.

#### 3) Intervention program

The design of the intervention has been described in detail elsewhere [17]. Briefly, all primary and high schools are required to conduct health education classes (routine health education) in China. Both control and intervention schools conducted their routine health education classes, while intervention schools additionally implemented the specially developed intervention components, comprising: a) classroom curriculum (including education on healthy eating and sufficient physical activity), b) school environment support, c) family involvement (including parents/guardians health classes), and d) fun programs/events. In full consideration of Chinese social convention, cultural and family traditions, and the existing primary education and examination system, these components were developed and integrated into the regular academic schedule of each intervention school.

### Data collection and measurement

Questionnaire surveys at baseline and immediately-post-intervention in May 2011(hereafter, follow-up) were administered by team members in the classroom with the assistance of a class teacher to collect information on students' demographic characteristics, students’ knowledge toward obesity and risk factors, eating behavior (frequency of intake of red meat and vegetables/fruits, consumption of high-dense-energy snacks and soft-drinks) and physical activity (PA) and sedentary behaviors (the time spent in TV viewing or computer use) in the last week. Parents/guardians were asked to report their families’ socio-demographic characteristics (including parental education, family size and structure) through a short questionnaire.

Students’ body weight and height were measured to the nearest 0.1 kilograms and to the nearest 0.01 meter, respectively, according to a standard protocol [17]. Measures were taken twice and the mean of the two readings was used in the analysis.

### Key study variables

The effectiveness of this lifestyle intervention program was evaluated based on changes in several outcome variables between the baseline and follow-up surveys: (1) body mass index (BMI), the primary outcome variable, (2) behavior patterns (physical activity and sedentary behavior), (3) lifestyle patterns (dietary intake), and (4) obesity-related knowledge.

BMI and body weight status: BMI was calculated by dividing body weight (kg) by the square of height (m^2^). There were three sub-outcome indices used regarding BMI. (1) Change in mean BMI value. This was used to evaluate the difference in BMI change in follow-up and baseline between intervention and control groups. (2) Change in participants body weight status (non-obese or obese). The proportions of non-obese participants at baseline becoming obese at follow-up and obese subjects at baseline becoming non-obese at follow-up were compared between two groups. Each participant's body weight status was assessed according to the age- and sex-specific BMI reference data recommended for Chinese children by the Group of China Obesity Task Force [19]. (3) Having ≥ 0.5 kg/ m^2^ decrease in BMI (D-BMI). This study was designed to detect a BMI difference of at least 0.5 kg/m^2^ between intervention and control students after intervention, therefore participants were categorized according to whether or not they had a ≥ 0.5 unit BMI change [17].

Behavior patterns (PA and sedentary behavior): Changes in frequencies of selected PA mode, commuting mode (to/from school), and sedentary behavior mode between baseline and follow-up were also used to evaluate intervention effectiveness. The specific PA mode included jogging/running, walking, and ball playing. The frequencies of each PA mode were assessed with the validated Chinese version of the International Physical Activity Questionnaire (CHN-IPAQ) [20]. The change in commuting mode was defined as walking or riding a bicycle to and from school at follow-up compared to being driven/ridden by parents/guardians at baseline. Sedentary behavior referred to viewing TV/video or using a computer. Participants were categorized as having either an increase or no-increase in frequency for each specific PA mode and for sedentary behavior.

Lifestyle patterns (Dietary intake): The consumption of red meat, vegetables, snacks and soft-drinks were evaluated using items selected from a validated food frequency questionnaire (FFQ) [21], which were further categorized into two subgroups according to the intake frequency: a decreased consumption of red meat, snacks and soft-drinks *vs*. a non-decreased intake; an increased consumption of vegetables *vs*. a non-increased vegetable intake.

Obesity-related knowledge and attitudes: Participants’ knowledge about obesity-related risk factors were investigated using questions about the frequency of consumption of fatty meat and fried snacks, vegetables, soft drinks, and frequency of physical inactivity and prolonged screen time. All these variables were categorized as “Yes”, “No”, or “Do not know”. For example, the question about frequent consumption of fatty meat was “Do you know that frequent consumption of fatty meat can increase the risk of gaining excess body weight?” The change in awareness of obesity-related knowledge was assessed between baseline and follow-up. The internal consistency of this questionnaire was estimated through calculating Cronbach’s alpha coefficient. The Cronbach’s α was 0.6 showing a moderate reliability of this questionnaire in this study. Participants were classified into two groups: those who changed from unawareness at baseline to awareness at follow-up *vs*. those who showed no change (remained unaware) at follow-up. At baseline only 1.3% (15/1125) students did not know that inadequate vegetable consumption might increase the risk of weight gain, thus consequently this question was excluded from further analysis.

### Statistical analysis

Demographic, social and clinical characteristics of participants were summarized by treatment group. Normality test was conducted on participants' age, height, weight and BMI value at baseline using Kolmogorov-Smirnov method, showing each of them with normal distribution. Then, comparisons between children who completed baseline and were lost to follow-up were conducted using Student’s t-test (continuous variables) or the chi-square test (categorical variables). Continuous variables were summarized as mean (standard deviation) and categorical variables as frequency (percentage).

Main characteristics of participants were balanced at baseline within intervention and control groups [18], and outcome variables were classified into binary categories in this study. Intervention effects were assessed using multivariate logistic regression models with adjustment for the school-level clustering effects, participants' age, gender, baseline body weight and parents' educational attainment. Outcomes from regression models were reported as an effect estimate and 95% confidential interval (95%CI). The unit of analysis was an individual child. All analyses were performed using SPSS 21.0 (IBM Corp, Armonk, NY, USA).

## Results

### Participants’ selected baseline characteristics

**Fig 1:**
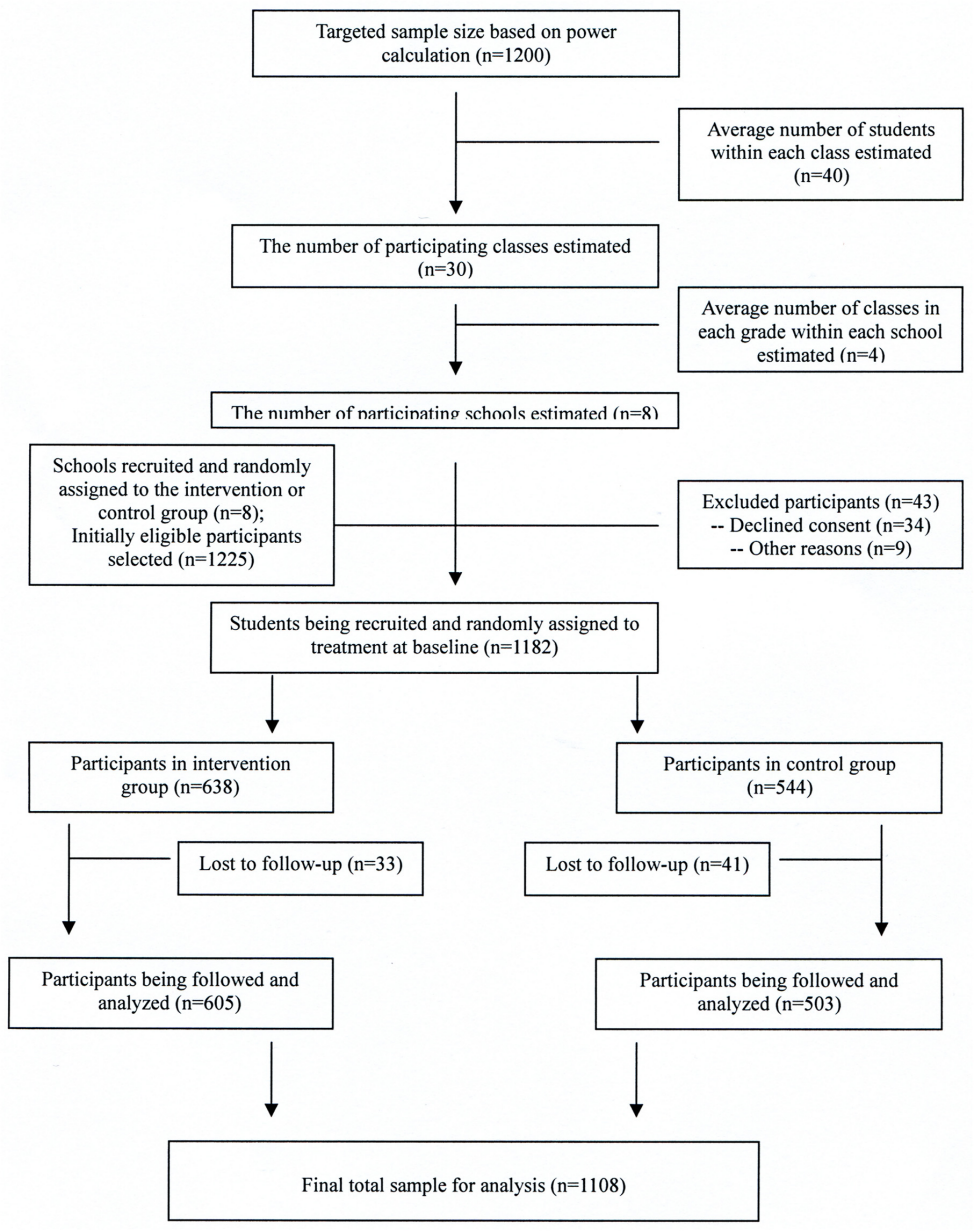
Flow diagram for enrollment and follow-up of students in the CLICK-Obesity Study.

Of the 1225 potentially eligible participants, 1182 (96.5%) agreed to participate in the trial at baseline, and 1108 (93.7% of participants) were successfully followed-up immediately after intervention. The main reasons for those lost to follow-up survey (n = 74) were that they were due to sickness or having other scheduled events on the survey day, which was evenly distributed in intervention and control groups. A participant flow chart of the trial is displayed in Fig 1. There were no significant differences in the percentage of children lost to follow-up between treatment groups (intervention *vs*. control = 33/638 *vs*. 41/544; *p* = 0.12), or between the baseline BMI of those followed-up and not followed-up (mean (±SD) BMI for followed-up *vs*. not follow-up = 18.6±3.1 *vs*. 18.3±2.9; *p* = 0.44). Table 1 displays the baseline demographic, social and clinical characteristics of the participants. The mean BMI at baseline was 18.54±2.92 and 18.71±3.17 (p = 0.36) for students in control and intervention group, respectively. There was no observable adverse event in the intervention group.

**Table 1:** Baseline demographic, social and clinical characteristics of study participants (4^th^ graders, n = 1108) by treatment group, Nanjing City, China.

		Treatment group†	
		Control (n = 503)	Intervention (n = 605)
Individual level			
	Mean (SD) age (years)	10.2±0.5	10.2±0.5
	Percentage of boys (95%CI)	58.3 (53.5, 62.6)	53.7 (49.6, 57.7)
	Mean (SD) weight (kg)	36.9±8.0	37.6±8.5
	Mean (SD) height (meters)	140.6±6.4	141.3±6.1
	Prevalence of obesity (%, 95%CI)	10.3 (7.9, 13.4)	11.6 (9.2, 14.5)
	Mean (SD) BMI	18.54±2.92	18.71±3.17
	Percentage of parents with educational attainment ≤9 years (95%CI)	24.1 (20.5, 28.1)	20.5 (17.4, 24.0)
Cluster level‡			
	Students per cluster	126	151
	Classes per cluster	4.0	4.5

†Comparisons between two groups in the variables were conducted using either Student’s t-test (continuous outcomes) or the chi-square test (categorical outcomes), showing no significant difference for each variable.

‡ The cluster level referred to school level.

### Intervention effects on BMI and obesity

More students in the intervention group than their counterparts in the control group had reduced BMI by 0.5 kg/m^2^ or more (46.0% *vs*. 39.0%; OR = 1.44, 95%CI = 1.10, 1.87), despite neither the mean of BMI (18.39±3.3 *vs*. 18.25±3.2, p = 0.43) nor the changes of BMI post intervention (-0.32±1.36 *vs*. -0.29±1.40, p = 0.09) between the intervention and control groups being statistically significant (Table 2). There was also no significant difference between intervention and control groups in the proportion of students who changed their body weight status either from baseline non-obesity to end-point obesity (intervention *vs*. control = 2.1% [9/438] *vs*. 2.2% [8/369]; *p* = 0.89) or from baseline obesity to end-point non-obesity (intervention *vs*. control = 28.7% [48/167] *vs*. 26.9% [36/134]; *p* = 0.82).

**Table 2:** Intervention effectiveness: height, weight and BMI at follow-up among 1108 primary school students in Nanjing City, China^^^.

Obesity		Treatment group		Mean difference†	95%CI	*P*
		Control	Intervention			
		(n = 503)	(n = 605)			
Height (cm)		148.17	149.00	0.83	0.01, 1.64	0.04
Weight (kg)		40.41	41.22	0.81	-0.32, 1.95	0.16
BMI						
	Mean value	18.25	18.39	0.14	-0.24, 0.53	0.43
	Changes ‡	-0.29	-0.32	-0.03	-0.18, 0.14	0.09

^The intervention lasted for two semesters from September 2010 to June 2011

† calculated from mean values at follow-up (intervention–control).

‡ calculated from BMI values at study end (follow-up–baseline); the intervention group had more BMI deduction than the control, which was marginally significant.

### Intervention effects on lifestyle behavior patterns

Table 3 presents outcomes regarding the lifestyle behaviors. Compared to the control group, the intervention group was more likely to increase the frequency of jogging/running (OR = 1.55, 95%CI = 1.18, 2.02), and reduce screen time (OR = 1.41, 95%CI = 1.09, 1.84) and red meat consumption (OR = 1.50, 95%CI = 1.15, 1.95). Among those students who commuted to and from school using a sedentary mode (by bus/city metro or driven by parents) at baseline, participants in intervention schools were more likely to change their commuting mode to walking or riding bicycles to school (OR = 2.24, 95%CI = 1.47, 3.40) relative to those in the control schools.

**Table 3:** Intervention effectiveness: changes in selected lifestyle behaviors among 1108 primary school students in Nanjing City, China^†^.

Factors influencing obesity	Change in frequencies of influence factors		
	% (n)		OR (95%CI)*
	Control (ref.) N = 503	Intervention N = 605	
**Improved physical activity patterns (increase in participating frequency)**			
Jogging/running	32.4 (163)	46.0 (278)	1.55 (1.18, 2.02)
Walking	45.7 (230)	46.9 (284)	0.98 (0.74, 1.25)
Ball playing	35.8 (180)	40.0 (242)	1.21 (0.93, 1.58)
**Improved commuting mode (from sedentary to active mode within students with sedentary mode at baseline)**			
Walking or riding bicycles to school ‡	16.5 (49)	28.9 (104)	2.24 (1.47, 3.40)
**Reduced viewing/using frequency**			
TV or computers	41.0 (206)	49.4 (293)	1.41 (1.09, 1.84)
**Change in dietary patterns** **			
Red meat	35.0 (176)	46.1 (279)	1.50 (1.15, 1.95)
Fried snacks	27.4 (138)	29.1 (176)	1.08 (0.81, 1.44)
Soft drinks	28.2 (142)	26.4 (160)	0.89 (0.67, 1.19)
Vegetables	47.1 (237)	48.6 (294)	1.20 (0.92, 1.55)

† Change in frequency was defined as participants whose physical activity frequencies increased and unhealthy food intake frequencies decreased at the follow-up.

‡ for control group, N = 297; for intervention group, N = 360

* Odds ratios were estimated with control group as the reference and using multivariate logistic regression methods with adjustment for clustering effect at school level, participants' age, gender, baseline body weight and parents' educational attainment.

** Change in red meat, snacks and soft-drinks presented as reduced consumption frequency, while in vegetables as increased frequency.

### Intervention effects on awareness of obesity risk factors

Table 4 shows changes in the awareness of five selected risk factors for obesity. Among students who were not aware of the five selected obesity risk factors at baseline, more participants in the intervention group than in the control group became aware of them at follow-up.

**Table 4:** Intervention effectiveness: changes in awareness of selected risk factors for obesity among students who were not aware of those at baseline in Nanjing City, China^†^.

Risk factors for obesity	Change in awareness (%) of risk factors (from ‘NO’ to ‘YES’)		
	% (N^‡^)		OR (95%CI)*
	Control (ref.)	Intervention	
Physical inactivity	64.7 (102)	83.7 (92)	2.70 (1.25, 5.87)
Prolonged screen time	27.8 (352)	65.8 (403)	5.16 (3.66, 7.27)
Frequent consumption of fatty meat	43.4 (205)	68.3 (183)	2.16 (1.33, 3.48)
Frequent intake of fried snacks	64.3 (168)	79.7 (187)	2.03 (1.22, 3.39)
Frequent intake of soft drinks	39.6 (260)	71.6 (320)	3.91 (2.67, 5.71)

† Change in awareness was defined as participants who did know the selected factors could increase the risk of gaining excess body weight at follow-up while they did not know at baseline.

‡ N, number of participants who were not aware of risk factors for excess body weight at baseline.

* Odds ratios were estimated with control group as the reference and using multivariate logistic regression methods with adjustment for clustering effect at school level, participants' age, gender, baseline body weight and parents' educational attainment.

## Discussion

Our study assessed the feasibility and effectiveness of a school-based childhood obesity-prevention lifestyle intervention program (CLICK-Obesity) within the current context of Chinese education and culture systems. We found that the intervention program significantly increased participants' healthy lifestyle behaviors and obesity-related knowledge. Compared with the control group, the intervention group was more likely to achieve a ≥0.5 kg/m^2^ BMI reduction (p<0.05).

Our findings suggest that the current school-based intervention program was feasible and effective in increasing leisure-time physical activity, improving active commuting mode, shortening screen time use, decreasing fatty meat intake, and advancing anti-obesity related knowledge for school students.

Schools are critical settings for preventing childhood obesity through the promotion of lifelong healthy eating and physical activity [6, 22]. To date, the majority of childhood obesity prevention programs have been conducted within schools, with many of these programs focusing on obese rather than non-obese children [8, 23, 24, 25]. However, it is of particular importance from a public health perspective to shift the entire distribution of risk among the general population, since such a shift can help reduce obesity prevalence and its associated burden at the broader population level. This study was specifically designed to meet this need in the area of childhood obesity prevention through the delivery of a lifestyle intervention in a general adolescent population.

Prevention of excess body weight gain can be achieved through two lifestyle modification approaches: (1) healthy eating, and (2) sufficient physical activity. Children are encouraged to adopt healthy eating, including consuming less high-energy density foods such as fatty meat, fried snacks and soft-drinks, while physically active lifestyle patterns are also strongly advocated for adolescents, including increasing time in moderate-to-vigorous physical activity and decreasing time in sedentary behaviors like TV viewing and computer use. These two approaches are widely used, either individually or together, in lifestyle interventions for obesity prevention in adults and children worldwide [5–7]. Healthy eating and sufficient physical activity are not only of particular importance for childhood obesity prevention but also of importance for children's body growth and development.

It is difficult to compare our study results with other similar studies due to different strategic components, follow-up periods, settings and social contexts. According to the 2013 U.S. Agency for Healthcare Research and Quality (AHRQ) report, the most systematic review on childhood obesity intervention programs to date, mixed results in school-based prevention studies have been observed to date. Some of the studies obtained significant changes in body weight status and its related variables [9]. Among the 124 school-based obesity intervention studies included in AHRQ report, only 21 were RCTs but none of these were from China [9]. Furthermore, only four of the 21 RCTs studies were the same as our study in terms of study design (RCT), settings (primary schools with family involvement), intervention components (healthy eating and physical activity), follow-up period (one year or less), and outcome assessment (changes in body weight or related measurement) [9]. However the results were still mixed from these four studies, with one showing no significant effectiveness [26] and three presenting results favoring the intervention [27–29].

Considering the significant changes in key outcomes that were reported after only a one year intervention, this study exceeded our expectations. Our study was specifically designed to develop a practical and effective population-based lifestyle and behavior intervention program aiming to prevent excess weight gain in the general population of school children in China. The lessons and experience from this study can provide important implications for future studies and intervention programs, for example:

1. Schools’ support and positive involvement is crucial. Prior to implementing this program, we consulted experts within local educational authorities, school principals and teachers to get their ideas and suggestions on the potential study. Based on their suggestions and strong support, we successfully integrated the four intervention components into the current educational system with minimum interference to participating schools’ normal academic programs.
2. Families’ strong support is needed. We also successfully obtained strong support from participants’ families. We received a clear and positive response to parents’ major concerns on whether or not the intervention components may exert a negative influence on their children’s body growth and development when they are at a body growth stage. Then, through a specifically developed health education class program, parents/guardians were educated to adopt healthy lifestyles and behaviors at home and asked to encourage their children to follow healthy lifestyle and behavior guidelines at home. The specific interactive events/activities were designed to ask students and parents to complete home assignments regarding healthy lifestyle and obesity prevention together. For example, students and parents were asked to measure body weight and height and then calculate the BMI for each other at home.
3. Fun events are of particular help. In addition to a widely used classroom curriculum, the sub-component of fun events was designed to be attractive and interesting for participants and readily incorporated into their normal academic schedule. For example, competitions for picture painting, short paper writing and stage drama were held regarding obesity and its associated risk to health, and healthy lifestyle and behaviors to combat obesity in daily life. Students were encouraged to tell/show their own stories about healthy diet and physical activity behaviors. We developed the intervention activities based on careful consideration of Chinese cultural and family traditions, social conventions, and the current primary education and examination system, which was the key factor in the success of this project.

This study has several strengths. First, it was designed to integrate with the current educational and social system operating in Mainland China and was supported by relevant findings from a similar school-based study conducted in the U.S. by one of our key team members, the HEALTH-KIDS study [30]. Second, multiple-components were integrated into each school’s regular academic programs. Furthermore, positive support was obtained from the parents of students and participating schools.

This study also has some limitations. First, we did not observe a significant reduction in overall mean BMI in the intervention group compared to the control group, although we observed some other desirable BMI-related changes. We suspect more significant desirable changes in BMI outcomes might have been observed if we could have included a larger study sample and our intervention and follow up was extended to a longer timeframe. To overcome such limitations, using similar lifestyle intervention strategies and lessons from this study we have since conducted a larger intervention program (The Health Legacy Project of the 2nd Summer Youth Olympic Games) which included more than 10,000 grade 4 and grade 7 children in Nanjing. This study has just been recently completed. Second, lifestyle and behavior patterns were self-reported, which might yield recall bias; and the questionnaires used to collect information on selected foods and knowledge were not specifically validated for Chinese adolescences. Third, the actual number of participants was less than the estimated sample size, which might be underpowered and reduced the ability to detect differences between groups. Fourth, the influence of the intervention on potential changes in parents' awareness of obesity-related knowledge and behavior was not assessed as the study focus was on the school children only. Fifth, the impact of school and family support and fun events on the success of this study was not specially examined.

In conclusion, the CLICK-Obesity study was found to be not only feasible but also effective in improving healthy lifestyle and behavior patterns and obesity related knowledge among primary school students through the delivery of a lifestyle intervention within schools in a large urban setting in China. The study provides important policy implications for future school-based childhood lifestyle modification programs in China and other developing countries.

## Supporting Information

S1 CONSORT Checklist CONSORT Checklist. (DOC)

S1 Protocol Trial Protocol in English. (DOC)

S2 Protocol Trial Protocol in Chinese. (PDF)
