# Supplementary material for: Effectiveness of a Randomized Controlled Lifestyle Intervention to Prevent Obesity among Chinese Primary School Students: CLICK-Obesity Study
Source: PLoS One. 2015 Oct 28;10(10):e0141421. doi: 10.1371/journal.pone.0141421 (PMC4625022; doi:10.1371/journal.pone.0141421)
Supplement: S2 Protocol — (PDF) [file pone.0141421.s003.pdf]

4、项目成果提交形式（发表论文篇数、实物成果形式、预期成果水平）

本项目成果提交形式为公开发表的专业论文：

在国际期刊发表1－2篇英文（SCI收录）论文，在国家级期刊发表3篇中文论文。

预期成果水平：

国内领先、国际先进。

四、项目研究方法和技术路线

技术方案：

设计方法：

学校为基础的随机对照干预（Cluster-Randomized Controlled Trials，RCT）随访研究。研究对象：9周岁（小学四年级）在校学生。

样本量：

计算效率基于本项目研究主要结局变量BMI值。研究目的是观察到BMI变化差异在干预组和对照组之间至少达到0.5kg/m²，且估算标准差为3.0 kg/m²。为推算整群设计研究的设计效率，依据学校和班级的规模，我们估算平均每所参与学校的样本量为150人，且基于以

往国外研究，组内相关系数（ICC）= 0.005，因此设计效率为 1.75。故我们推算若来自这 8 所学校的 1200 名学生完成本项目，将会有 80% 的把握度观察到干预组和对照组之间的 BMI 变化差异（α 单侧= 0.05）。

抽样方法：

在建邺区采用多阶段随机整群抽样方法选择 8 所小学；在学校水平进行随机分组，即所抽取学校的全部四年级学生均作为研究对象。项目正式实施前，所有项目学校、研究对象和家长均需签署知情同意书。

问卷调查：

所有调查内容设计成统一的标准调查表，采用面对面调查询问的方式，对调查对象进行基线调查和干预 1 年后的评估调查。研究内容主要包含社会人口学信息、家庭信息、生活学习相关信息、饮食（类型和频率）、体力活动（频次和数量）、相关的 KAP。分别在基线和随访时点测量研究对象的身高、体重。体检地点统一在学校指定地点，由专业技术人员进行收集。

体格测量：

采用标准方法和仪器准确测量身高和体重。身高精确到 0.1cm，体重精确到 0.1 kg。以班级为单位逐一测量，每种人体测量指标均测量 2 次，取平均值。指标测量由各学校经过统一培训和考核的保健

医师专人负责。干预 1 年后，由相同的体检人员采用同一测试方法和同一型号仪器、在同一地点和时间检测干预组与对照组学生的身高和体重。

干预方法和内容：

（1）干预方法：干预措施以打包（Package）的形式整体实施，通过课堂健康教育课程（合理膳食和充足体力活动）+学校环境支持+家庭帮助+兴趣项目等综合性方法对干预组学生开展相关内容和主题的干预活动；对照组除常规健康教育和体育课外，不采取任何干预措施。（2）主要干预内容包括：①课堂健康教育课程：传播知识和技能以促进学生合理膳食和增加校内外体育活动时间。每个月（8 月/学年）由经过培训过的班主任统一授课 1 次（30 分钟）。内容包括肥胖主题 1 次、健康膳食主题 4 次和充足体力活动主题 3 次。项目组成员现场参与每次听课。②学校环境支持：健康相关的信息和海报张贴在干预学校的教室、体育馆、操场和食堂等公共场所。结合规定的干预主题，每月更新班级黑板报。③家庭帮助：举办两次家长会，由班主任组织、项目组成员进行肥胖干预项目介绍，鼓励家庭学习、参与、探讨。第一学期介绍肥胖的危害、健康膳食与家庭合理膳食氛围的营造；第二学期介绍充足体力活动的意义、居家的健康生活行为方式。并且学生与父母共同完成健康膳食与充足体力活动相关的家庭作业。④兴趣项目：1. 两学期举办绘画、小作文和舞台剧等 3 次竞赛，内容包括肥胖及其健康危害、抵御肥胖的健康生活行为方式。2. 开展个人行为互动实践活动，包括“无快餐周”、“无电视周”、“无软饮料周”。

数据处理：

使用 Epidata 软件编制和录入数据，使用 SPSS 软进行数据整理和分析。应用 t-test 和 x2 分析方法进行有关均数和比例的比较，使用 Logistic 和线性回归分析方法进行相关因素间的联系以及效果评估的统计学分析。

随访与质控：

随访：

以课题组成员为核心组成固定的随访小组实施为期一年的现场综合干预，于干预后 1 年进行随访评估干预效果；随访内容格式化；失访率控制在 10%以内。

质控保障：

1. 使用成熟的调查表；
2. 调查表经过验正；
3. 课题组成员作为调查员，且经过统一的系统专项培训；
4. 调查点均安排质控人员进行现场质量监控，要求调查表填写客观、完整；
5. 基线调查的有效率大于 90%、保证样本的代表性；
6. 干预措施整体化、定量化；
7. 体检由临床医务专职员负责实施；
8. 调查表数据由两组人员独立双份录入并核对及更正。
