# Supplementary material for: Effectiveness of a Randomized Controlled Lifestyle Intervention to Prevent Obesity among Chinese Primary School Students: CLICK-Obesity Study
Source: PLoS One. 2015 Oct 28;10(10):e0141421. doi: 10.1371/journal.pone.0141421 (PMC4625022; doi:10.1371/journal.pone.0141421)
Supplement: S1 Protocol — (DOC) [file pone.0141421.s002.doc]

Dear Editor,

Below is the translation of section 4, the study protocol from this study’s proposal. The original is in Chinese and has been scanned as pdf document and uploaded too.

Please do not hesitate to let me know if you have any query.

Many thanks.

Fei Xu

儿童生活行为方式的综合干预：肥胖的预防

Comprehensive Lifestyle Intervention among Chinese Kids: Obesity Prevention （CLICK-OBESITY）

**SECTION 4. Study Protocol**

**Study design:**

School-based Cluster-Randomized Controlled Trials. Participants are grader 4 (9 years old) school students in primary schools.

**Sample size:**

The sample size was estimated based on the primary outcome variable, BMI. We would like to observe minimum 0.5 kg/m^2^ difference in BMI between control and intervention group, considering a standard deviation of 3.0 kg/m^2^. To estimate the design coefficient due to cluster sampling approach used in this study, we investigated the registered students’ number being approximately 150 on average in each grade within local primary schools, and adopted intraclass correlation coefficient (ICC) as 0.005 based on previous similar studies conducted in other countries. Then, we calculated that the design coefficient was 1.75. Therefore, we estimated that approximately 1200 participants from 8 primary schools might be sufficient for us to detect the change in BMI between these two groups with 80% statistical power based onα= 0.05 (one-side).

**Sampling method:**

Multi-stage sampling method was used to randomly select 8 primary schools from Jianye district. The randomization was at school level. All grade 4 students within chosen schools will be the eligible participants. Prior to implement, all participating schools, students and their parents/guardians will be asked to sign the written informed consent.

Questionnaire survey

There will be two rounds surveys, the baseline and follow-up (after intervention) survey. All the information, integrated into one questionnaire, will be collected through face-to-face interview. The information gathered are regarding socio-demographic characteristics, school learning, dietary (type and frequency), physical activity (frequency and duration), related and KAP.

Participants’ body weigh and height will be measured by trained professionals within designated places at baseline and follow-up, respectively.

**Physical examination:**

Standardized approaches will be used to measure students’ body weight and height. The weight will be measured to the nearest 0.1 kilograms and height measured to the nearest 0.1centimeter. Each student will be measured twice and the mean value will be used for analysis. This examination will be applied to students class by class.

The trained and tested healthcare teachers will be responsible for the physical examination. After one-year intervention, the same trained healthcare teachers will conduct the physical examination for students, with the same approach and same type instruments, in the same place.

**Intervention components:**

(1) Intervention approach

All the intervention components will be packaged through classroom curriculum (healthy eating and sufficient physical activity), school environment support, family involvement and fun events to build a comprehensive intervention strategy, delivering to all students within intervention schools. The students within control group will receive their routine health education and exercise program regulated by government, but get no such intervention program.

(2) Intervention components

①Classroom curriculum: To disseminate knowledge and skills of healthy eating and promotion of physical activity time on- and off-campus, the 30-munite curriculum will be delivered monthly (8 times per semester year) by trained class head teachers. The contents include obesity (1 time), healthy eating (4 times) and sufficient physical activity (3 times). The team member will audit each class.

②School environment support: Health related posters will be put up in classrooms, gyms, playgrounds and students’ canteen. Each class will monthly update the content of billboard according to the intervention components.

③Family involvement: Two parents/guardians class will be held by class head teachers and team members will disseminate information on this project and encourage family positively to take part in this project. The first parents class will focus on risks of obesity, healthy eating and how to build family atmosphere for healthy eating. The second class will target sufficient physical activity, including benefit of sufficient physical activity, physically active lifestyle at home. Parents will be invited to do some home assignment regarding healthy eating and sufficient physical activity.

④Fun events: First, Three competitions (panting, short paper writing and stage drama) will be held regarding hazards of obesity, healthy lifestyles against obesity. Second, the self-administered “healthy week” will be scheduled sticking to “No snacks”, “No TV viewing”, “No soft drinks”.

**Data analysis:**

The Epidata software will be sued to enter data and SPSS will be used to manage and analyze data. T-test and Chi-square approaches will be applied to compare the difference in means and proportions between two groups. The regression models (Logistic and linear) will used to investigate the associations between factors and to assess the effects of intervention.
